# Comprehensive Quality Assessment Based Specific Chemical Profiles for Geographic and Tissue Variation in *Gentiana rigescens* Using HPLC and FTIR Method Combined with Principal Component Analysis

**DOI:** 10.3389/fchem.2017.00125

**Published:** 2017-12-22

**Authors:** Jie Li, Ji Zhang, Yan-Li Zhao, Heng-Yu Huang, Yuan-Zhong Wang

**Affiliations:** ^1^Institute of Medicinal Plants, Yunnan Academy of Agricultural Sciences, Kunming, China; ^2^College of Traditional Chinese Medicine, Yunnan University of Traditional Chinese Medicine, Kunming, China

**Keywords:** *Gentiana rigescens*, HPLC, FTIR, principal component analysis, quality assessment

## Abstract

Roots, stems, leaves, and flowers of Longdan (*Gentiana rigescens* Franch. ex Hemsl) were collected from six geographic origins of Yunnan Province (*n* = 240) to implement the quality assessment based on contents of gentiopicroside, loganic acid, sweroside and swertiamarin and chemical profile using HPLC-DAD and FTIR method combined with principal component analysis (PCA). The content of gentiopicroside (major iridoid glycoside) was the highest in *G. rigescens*, regardless of tissue and geographic origin. The level of swertiamarin was the lowest, even unable to be detected in samples from Kunming and Qujing. Significant correlations (*p* < 0.05) between gentiopicroside, loganic acid, sweroside, and swertiamarin were found at inter- or intra-tissues, which were highly depended on geographic origins, indicating the influence of environmental conditions on the conversion and transport of secondary metabolites in *G. rigescens*. Furthermore, samples were reasonably classified as three clusters along large producing areas where have similar climate conditions, characterized by carbohydrates, phenols, benzoates, terpenoids, aliphatic alcohols, aromatic hydrocarbons, and so forth. The present work provided global information on the chemical profile and contents of major iridoid glycosides in *G. rigescens* originated from six different origins, which is helpful for controlling quality of herbal medicines systematically.

## Introduction

For centuries, *Gentianae Radix et Rhizoma* (Longdan in Chinese) has been prominent in treating liver diseases in the traditional Chinese medicine system, one of origins of which is the roots or rhizomes of *Gentiana rigescens* Franch. ex Hemsl (State Pharmacopoeia Commission, 2015). *G. rigescens* (GR) belonging to Gentianaceae family, with the Chinese name of Dianlongdan or Jianlongdan, is a perennial herb native to Yunnan Province, China (Editorial Committee of Flora China Nica of the Academy of Sciences of China, 1988). In addition to the document in Chinese Pharmacopoeia, a wealth of clinical experience from Yi ethnic minority group verified its treatment uses of hepatitis and cholecystis (Suyama et al., 2017). The major compounds in GRs include iridoid glycosides and xanthones (Pan et al., 2016), active ingredients responsible to various pharmacological effects such as anti-inflammation, antioxidant, anti-cancer, antiviral, and so forth (Ma et al., 2001; Wang et al., 2013; Guo et al., 2014; Fabiani, 2016; Balkan et al., 2017). Iridoid glycosides belong to the monoterpene family and are divided into iridoid glycosides and *seco*-iridoid glycosides, depending on the bond between C-7 and C-8 of cyclopentene skeleton. GRs typically contain three *seco*-iridoid glycosides and one iridoid glycoside: gentiopicroside, sweroside, swertiamarin, and loganic acid (Pan et al., 2015). A number of studies have revealed that these four ingredients, and especially their derivate compounds, function as agents against hepatitis, inflammation, bacteria, free radical, etc. (Kumarasamy et al., 2003; Suyama et al., 2013; He et al., 2015). For example, gentiopicroside act the hepatoprotective effect by changing bile acids metabolism to correct the dyshomeostasis induced by pathogen (Tang et al., 2016), while the swertiamarin and sweroside instead by enhancing the activities of superoxide dismutase and catalase and the level of glutathione (Mihailović et al., 2014). Compounds with bioactive effects have been increasingly important to the quality regulation of herbal medicines in recent years, due to properties of inherence and abundance in favor of detection (Liu et al., 2016; Ding et al., 2017).

It has been widely recognized that environmental factors appear to play important roles in the accumulation of secondary metabolites in plant tissues, a consequence of that adjusting the growth environment may change the transcription and expression of genes related to the biosynthesis (Zoratti et al., 2014; Vuorinen et al., 2015). Zanatta et al. (2017) investigated the phytochemicals in soybeans cultured in two microregions, the result of which indicated that contents of protein, fatty acid, total carotenoid and γ-tocopherol were higher in microregion II where the main features are the relatively low altitude and precipitation. Melito et al. (2016) suggested that chemical profiles of *Helichrysum italicum* grew in seaside and mountains were different, particularly when plants lived in the farther climatic locations was diverse. The Yunnan area is characterized by the three-dimensional climate on low latitude plateau, where a changing climate and complex terrain have been created naturally as a place for displaying the plant diversity. The strong adaptive capability to different environmental conditions makes GR become a widespread species in Yunnan area, with three kinds of climatic cones i.e., tropical, subtropical, and temperate zones, large scope of altitude, annual average temperature and annual precipitation, and various soil types (Zhang et al., 2012). Maintaining the homogeneous quality of GRs, of which original plants live in complex growth condition, is a challenge. Thus, the quality control and assessment is paramount importance. It is not clear that how the geographical factors influence the content of secondary metabolites in GR, not just gentiopicroside, despite Wu et al. (2017) establishing an evaluation strategy for discriminating GRs collected from different geographical origins by FT-IR coupled with PLS-DA and SVM-GS.

Methods that can reflect the chemical features and accurately quantify target compounds in samples is critical to the quality control and assessment of herbal medicines. Providing the convenience for both qualitative and quantitative analysis, high performance liquid chromatography (HPLC) in combination with different spectrometric detectors has traditionally been the workhorse of herbal medicines' studies (Song et al., 2013). Zhang et al. (2016) simultaneously determined nine active ingredients isolated from *Salvia miltiorrhiza* and its variety using HPLC-DAD method, followed by revealing latent difference in components via principal components analysis (PCA) and partial least squares discriminant analysis (PLS-DA). One of the most widely utilized techniques to obtain information of chemical structures is Fourier transform infrared spectra (FTIR), especially when combined with pattern recognition methods (Hirri et al., 2016). The most useful chemical constituents for diagnostic monitoring of storage effects on quality control of traded saffron was achieved using FT-MIR coupled to PCA, according to the study by Ordoudi et al. (2014). Moreover, other tools that have found considerable success in herbal medicines uses are gas chromatography coupled with mass spectrometry (GC-MS), ultra-performance liquid chromatography-quadrupole time of flight mass spectrometry (UPLC-QTOFMS), high performance thin-layer chromatography (HPTLC) and so on (Nguyen et al., 2016; Guzelmeric et al., 2017; Liu J. et al., 2017).

In the present work, the quality assessment of GR samples collected from six geographic origins of Yunnan area was carried out using HPLC combined with IR rather than other expensive techniques, meanwhile, in conjunction with the PCA. Gentiopicroside, sweroside, swertiamarin, and loganic acid were selected to be standard compounds for the quantitative analysis of chromatography. This study may provide some potential supports on the reasonable application and exploitation of GR.

## Materials and methods

### Chemicals and plant materials

The extraction solvent (methanol) and potassium bromide (KBr) were purchased from Tianjin Fengchuan Chemical Reagent Technolodies Co., Lid. (Tianjin, China). Acetonitrile and formic acid (HPLC grade) were obtained from Thermo Fisher Scientific (Fair Lawn, NJ, USA) and Dikmapure (Lake Forest, CA, USA), respectively. Purified water was provided by Hangzhou Wahaha Group (Huangzhou, China). All other chemicals and reagents were analytical grade. The standard compounds (**1**, loganic acid; **2**, swertiamarin; **3**, gentiopricroside; **4**, sweroside) were purchased from the National Institutes for Food and Drug Control (Beijing, China). Purities of standard compounds were all > 98%.

One hundred and seventy eight specimens of *G. rigescens* (Gentianaceae) were collected from 15 different sites characterized by varied environmental conditions in Yunan and Guizhou Provinces, China (Table 1). The root, stem, leaf, and flower of each individual were separated. All of samples were authenticated by Prof. Hang Jin (Institute of Medicinal Plants, Yunnan Academy of Agricultural Sciences, Kunming, China), and voucher specimens (DLDYS20121112 and DLDYS20131011) were deposited at the specimen room of Institute of Medicinal Plant, Yunnan Academy of Agricultural Sciences.

**Table 1:** Collected information of samples.

**Number**	**Source**	**Environment**	**Elevation/m**	**Latitude**	**Longitude**	**Time**
**YUNNAN PROVINCE**						
P45_WY 1-10	Cangshan Global Geopark, Dali	—	2225	N25°39′03.9″	E100°09′48.9″	24th November, 2012
P67_CY 1-10	Mopanshan Mountian, Xinping, Yuxi	—	2016	N23°58′01.1″	E101°56′57.1″	18th December, 2012
P71_CY 1-10	Asugou Village, Yiliang, Kunming	Pine woods	1817	N25°02′57.7″	E103°17′39.6″	28th December, 2012
P81_CY 1-10	Duoluotun Village, Fuyuan, Qujing	Weeds	2072	N25°42′22.1″	E104°10′27.5″	28th December, 2012
P52_NY 1-10	Shuicaoba Village, Ninglang, Lijiang	—	3219	N27°20′25.2″	E100°59′01.1″	6th December, 2012
P54_NY 1-10	Haini Village, Weixi, Diqing	—	2520	N27°31′05.5″	E99°22′10.3″	6th December, 2012
**GUIZHOU PROVINCE**						
P190 1-10	Guji Village, Hezhang Town	Fir woods	2018	N27°18′21.2″	E104°45′57.3″	10th October, 2013
P191 1-10	Huopu Town, Panxian	Fir woods	1983	N25°39′51.7″	E104°23′52.8″	24th, October, 2013
P199 1-11	Baota Mountian, Zhanjie Town, Qingzhen	Pine woods	1590	N26°38′22.6″	E104°43′26.0″	24th, October, 2013
P200 1-10	Daxingzai Village, Wusha, Xingyi	Pine woods	1598	N25°07′00.7″	E104°43′38.2″	5th November, 2013
P205 1-10	Longxiang Mountain, Longli, Duyun	Pine woods	1300	N26°27′38.7″	E106°55′40.8″	5th November, 2013
P197 1-20	Shaping Village, Huangsi Town, Fuquan	Weeds	1550	N26°34′10.0″	E107°21′53.9″	5th November, 2013
P198 1-18	Bailong Village, Kaiyang, Guiyang	Weeds	1600	N27°01′35.2″	E106°19′09.0″	24th, October, 2013
P204 1-10	Jichang Village, Longli, Duyun	Weeds	1393	N26°33′33.6″	E106°54′59.2″	5th November, 2013
P208 1-19	Hongyang Village, Taigong Town, Taijiang	Weeds	1260	N26°33′36.2″	E108°19′45.5″	5th November, 2013

### Sample preparation

Standard solutions were dissolved in HPLC grade methanol individually to achieve a stock with concentration of 3.9 mg/mL (gentiopicroside), 1.1 mg/mL (loganic acid), 0.7 mg/mL (sweroside), and 1.0 mg/mL (swertiamarin). The calibration standards were prepared at 11 levels from 0.003 to 1.9 mg/mL (gentiopicroside), 0.001–0.5 mg/mL (loganic acid), 0.001–0.3 mg/mL (sweroside) and 0.0009–0.5 mg/mL (swertiamarin) for establishment of external standard calibration curves. The calibration curve was obtained by plotting the chromatographic peak area of standard compounds (Y) vs. the corresponding concentrations (X). All standard compounds were stored at −20°C when not in use.

Root, stem, leaf and flower samples were dried at 50°C in a 101A electric thermostatic drying oven (Experimental Instrument Factory, Shanghai, China). Dried samples were milled and sifted through a 60 mesh sieve, respectively. An accurately weighted sample powder (0.025 g) was extracted by 1.5 mL 80% methanol for 45 min. The extract solution was filtered through 0.22 μm membrane filters. Then, the filters collected in auto sampler were analyzed directly by LC systems. 1.2 mg sample powder was mixed evenly 100.0 mg KBr crystal. The mixture was ground and pressed into a tablet.

### High performance liquid chromatography (HPLC) analysis

Analyses were performed on an Agilent 1260 HPLC system (Agilent Technologies, Santa Clara, California, USA) composed of a G1315D diode-array detector (UV-vis, DAD, 190-400 nm), a G1311C VL quaternary gradient pumps equipped with a vacuum degasser, a G1316A thermostatted column compartment and a G1329B ALS auto sampler. The modified version of our published HPLC method (Chu et al., 2016) was used as separation conditions in this study. The separation was carried out on Agilent Intersil-C_18_ column (150 × 4.6 mm, 5 μm). The mobile phase consisted of 0.1% formic acid in water (A) and acetonitrile (B). The samples were eluted with the following gradient: 0.0–0.4 min, 0–7% B; 0.4–2.5 min, 7–10% B; 2.5–20.0 min, 10–26% B; 20.0–29.0 min, 26–58.3% B; 29.0–30.0 min, 58.3–90% B; 30.0–34.0 min, 90% B. The flow rate was 1.00 mL/min. The injection volume was 10 μL. The detective wavelength was set at 246 nm. The temperature-controlled column oven was set at 30°C. Methanol and water supplemented 0.1% formic acid were degassed by ultrasonication for 30 min to avoid bubbles in solutions prior to analysis.

### Fourier transform infrared (FTIR) spectroscopy analysis

Infrared absorption spectra of samples were recorded using a FTIR spectrometer (Perkin Elmer, Foster City, CA, USA) equipped with a deuterted triglycine sulfate (DTGS) detector. Typically, the accumulation spectra of 16 scans per samples was collected and averaged. Absorption spectra in the area between 4,000 and 400 cm^−1^, at a resolution of one data point every 4 cm^−1^, were obtained. Interferences of CO_2_ and H_2_O in air were eliminated automatically.

### Method validation

For reference standards, linearity, the limit of detection (LOD), the limit of quantification (LOQ), precision and accuracy were experimentally verified. The LOD and LOQ, signal-to-noise ratios (S/N) of 3 to 10, were determined by serial dilution of each standard solution using the described conditions.

Precision was evaluated by intra- and inter-day variation determined by analyzing mixed standard solutions with known concentration six times within a day and on three consecutive days in triplicate. Accuracy was validated by recovery test that was performed by accuracy adding three different amounts (low, medium, and high spike) of the reference standards to the crude samples. The recovery rate was calculated as follow:

%R = [(measured amount – original amount) / amount added] × 100%.

### Data analysis

PCA were performed for assessing the difference of chemical constituents in samples from various geographic origins using FTIR data sets, which were second derivative-transformed before analysis. ANOVA followed by Tukey's test at *p* < 0.05 were employed to evaluate the statistical significance of differences in the variables among different samples. Correlations between gentiopicroside, loganic acid, sweroside, and swertiamarin were achieved by Pearson's correlation analysis. All assays were unfolded using R 3.4.0 program (R Core Team, 2017).

To assess possible correlations of chemical profiles with environment conditions, annual mean temperature, annual mean relative humidity, and annual mean precipitation data from 1981 to 2010 for the sampling sites of *G. rigescens* were downloaded from Climatic Data Center, National Meteorological Information Center, China Meteorological Administration.

## Result and discussion

### Quantitative analysis of four target compounds in GR

**Figure 1:**
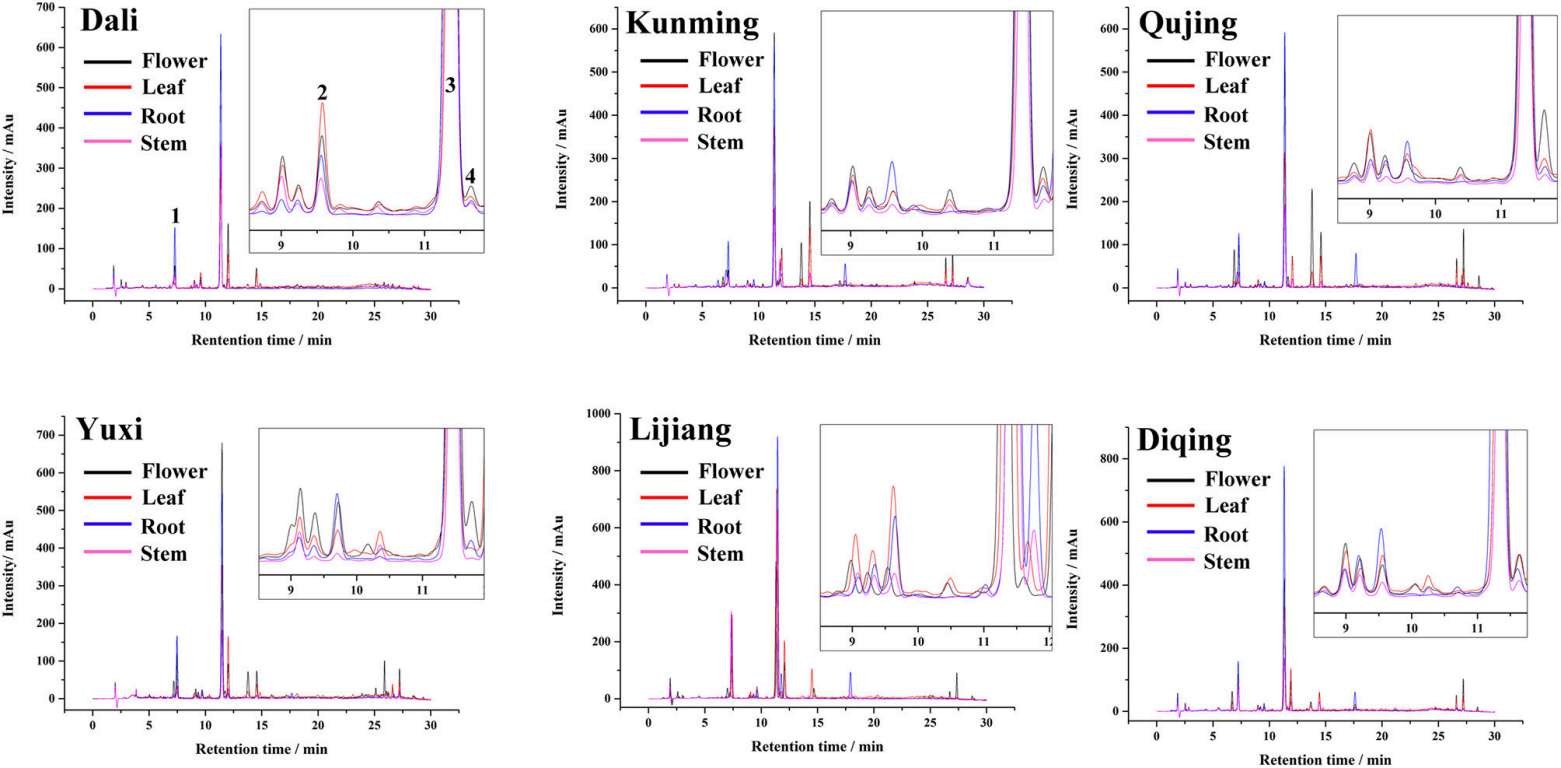
HPLC chromatograms plot of different tissue samples of *G. rigescens* from six geographic origins.

**Figure 2:**
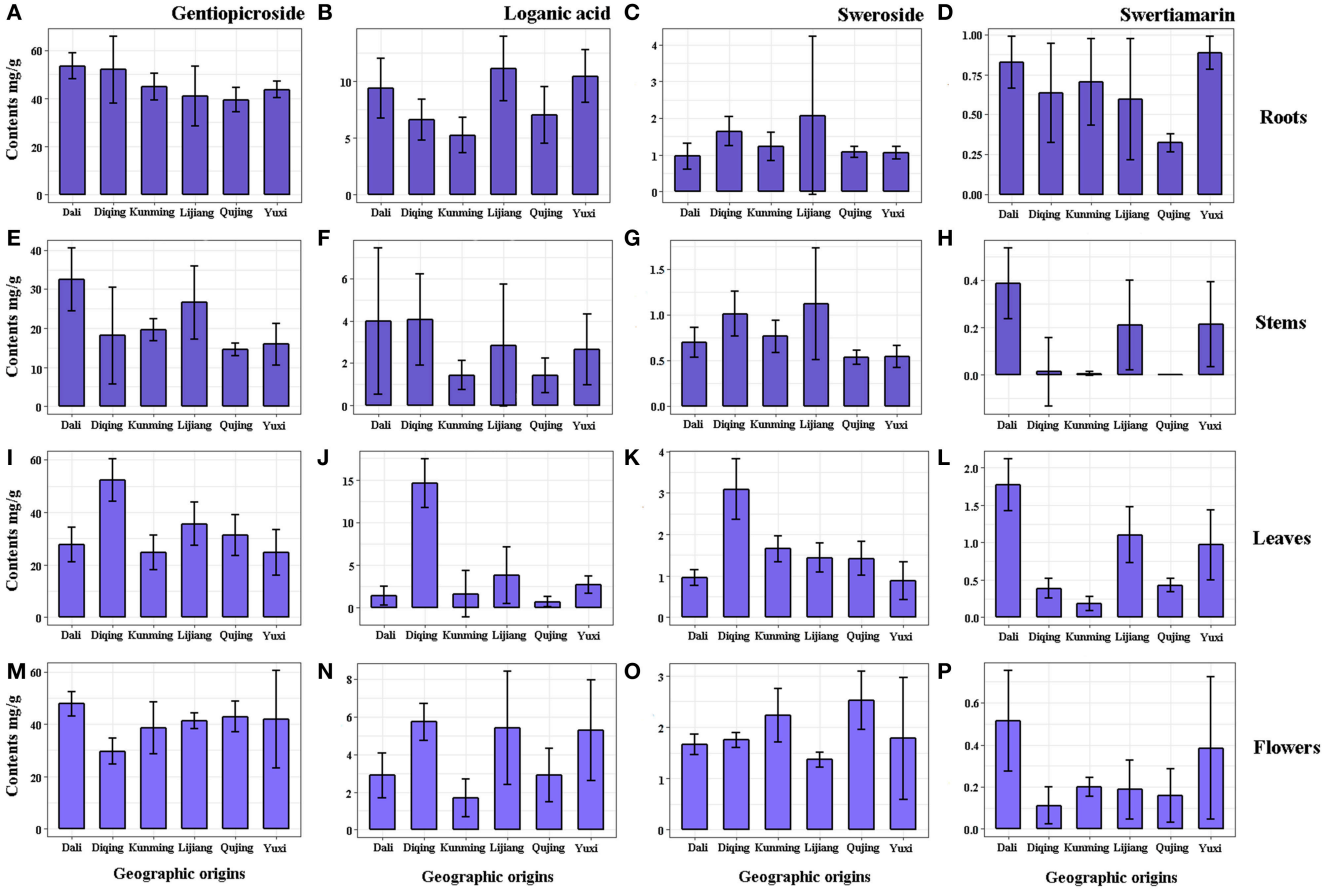
The contents of gentiopicroside, loganic acid, sweroside and swertiamarin in roots **(A–D)**, stems **(E–H)**, leaves **(I–L)** and flowers **(M–P)** of *G. rigescens* from six geographic origins (mg/g, *n* = 10).

Figure 1 shows the plot of chromatographic peaks of different tissue samples (roots, flowers, leaves, and stems) from six geographic origins. An appreciable amount of compounds were isolated under the gradient elution by HPLC-DAD, according to the chromatogram. Among them, four target compounds (peak 1–4) were identified unambiguously as loganic acid, swertiamarin, gentiopicroside, and sweroside, respectively, by comparing with the retention times of reference standards. The content of each compound was calculated by their calibration curve, and the result was shown in Figure 2 and Table S1.

From the results, gentiopicroside (14.55 ± 0.75–53.40 ± 2.34 mg/g) was the predominant ingredient in GR regardless of tissue types and geographic origins, of which the content was significantly more than other constituents. Of four tissues, the root maximized accumulations of gentiopicroside (39.41 ± 2.24–53.40 ± 2.34 mg/g) and loganic acid (5.24 ± 0.71–1.13 ± 1.23 mg/g). Remarkable variations (*p* < 0.05) were observed in gentiopicroside and loganic acid in tissues from six geographic origins, except for the root which has historically been regarded as the medicinal part for treating various ailments. Contents of gentiopicroside in roots from Dali and Diqing (Figure 2A) and in leaves from Diqing (Figure 2I) were significantly higher than that from other origins, while the low level of that was found in stems from Qujing (Figure 2E). The root still has been regarded as the main site to accumulate gentiopicroside despite the geographical factors changing the contents more or less, combined with the study performed by Qi et al. (2017a,b), which focused on the chemical diversity of GR on different cultivation years. Still, leaves from Diqing contained higher contents of loganic acid compared with others (Figure 2J). Leaves from Dali and Qujing showed significant low contents of this compound. The fairly low contents were found on the rest of two compounds in all samples, particularly the swertiamarin (< 1.78 ± 0.15 mg/g) which even could not be detected in some samples such as stems from Kunmig and Qujing (Figure 2H). Six origins in present work are geographically divided into three large producing areas: Dali is located in the western Yunnan characterized by the high precipitation (Figure S1A); Yuxi, Kunming, and Qujing lie on the central Yunnan featured as the high relative humidity (Figure S1B); Lijiang and Diqing are situated in the northwestern Yunnan famous with the low temperature (Figure S1C). From the tissue point of view, there were high contents of gentiopicroside, loganic acid, and sweroside in leaves from the cold northwestern Yunnan. Then, high levels of gentiopicroside existed in roots and flowers from western Yunnan with abundant precipitation. Combining content data described above, we speculated that the temperature and precipitation are two important factors influencing the accumulation of secondary metabolites. The temperature was the major factor influenced the accumulation of secondary metabolites in the leaf, whereas that in the root, stem, and flower was mainly depended on the precipitation. These results also indicated that secondary metabolites express a tight link to environmental factors, in which the high humidity showed an adverse effect on the accumulation of (*seco*-) iridoid glycosides.

Gentiopicroside is the major compound in *G. rigescens* as well as the unique indicator of quality assessment recommended by Chinese Pharmacopoeia. Our results showed that plants from Dali have the potential powerful pharmaceutical effects not only due to high content of gentiopicroside but also loganic acid, swertiamarin, and sweroside.

The total contents of four target compounds were calculated for eliminating the interference of tissues (Figure S2 and Table S1). Contents of gentiopicroside in whole plants from central Yunnan were significantly lower than other two big producing areas (Figure S2A), whereas the relatively high level of loganic acid was observed in samples from northwestern Yunnan with low temperature (Figure S2B). Enhanced water treatment decreased iridoid glycoside concentrations, according to the precipitation patterns established by Jamieson et al. (2013). Martz et al. (2009) indicated that low temperature favored higher contents of iridoids concomitantly reduced the content of flavonols in *Menyanthes trifoliate*. Also, Liang et al. (2014) reported the clear-cut inhibitory effect of high temperature on the synthesis of iridoid glycosides in *Scrophularia ningpoensis*. With strong support from these works, which could explain the relatively low content gentiopicroside from central Yunnan, the combination of multiple environmental factors should be considered as a vital role to influence the accumulation of iridoid glycosides in GR. Nevertheless, impacts of three climate factors on contents of sweroside and swertiamarin were not evident, although the low temperature of northwestern Yunnan caused slightly high and low levels of them, respectively. More studies are needed to unfold for compounds just like sweroside and swertiamarin using more sensitive techniques, because accumulative rules may be hidden by their small amounts.

### Chemicals-geographic origins association analysis

Quality definition of GR is generally depended on levels of getiopicroside whose accumulation was apparently distinct in samples from different geographical origins. Association analysis of target ingredients across specimens of inter- and intra-tissues provides the approach to further understand the influence of environmental factors on the quality of this herbal medicine. To address this issue, Pearson's correlation analysis was performed between contents of four major ingredients (i.e., gentiopicroside, loganic acid, sweroside, and swertiamarin) belonging to the biosynthesis pathway of (*seco*-) iridoid glycosides in Gentianaceae species (Li et al., 2017). Pearson's correlation analysis is based on the Pearson's correlation coefficient (PCC), a range of values from 1 (a perfect positive correlation) to −1 (a perfect negative correlation) with 0 representing a random distribution (Barlow et al., 2010), which is designed for describing the linear relationship of intensity distributions between two channels in pattern recognition (Zinchuk et al., 2005).

From the Table 2, the focus of association analysis in samples of Dali (western Yunnan) was the bond between target ingredients in intra-tissues, particularly in stems. There was a significantly positive correlation (*p* < 0.01) between loganic acid and sweroside in both leaves and roots from Dali. Biosyntheses of sweroside, gentiopicroside, and swertiamarin were found to rely on loganic acid whose creation mainly happens in leaves, as the precursor, according to most reports about (*seco*-) iridoid pathway in recent years (Miettinen et al., 2014; Tham et al., 2016) However, discussing in the study associated with *seco*-iridoid biosynthesis in *Swertia mussotii*, Liu Y. et al. (2017) indicated that gene expressions associated with synthesis were extremely low in roots except for one named *Sm7DLGT* responsible to the coding of 7-deoxyloganetic acid glucosyl transferase who catalyzes the reaction of producing 7-deoxyloganetic acid (loganic acid's precursor). Hua et al. (2014) also hypothesized that *seco*-iridoids were transformed to the roots after finishing the synthesis in other tissues. Even yet, relatively few is known about which organ the biosynthesis of intermediate products take place in *Gentiana* species. Therefore, we speculated that the correlation between loganic acid and sweroside tends to represent the conversion relationship in intra-tissue. Even leaf's advance for producing loganic acid is likely to attribute to its synthesizing factory role known as “source,” with our data showing that correlation between loganic acid and sweroside were significant in leaves, relationships between loganic acid and other compounds could be evident in stems. Loganic acid in stems as well as gentiopicroside, revealed highly positive correlations (*p* < 0.05) with both swertiamarin and sweroside which simultaneously played a positive correlation (*p* < 0.05) with its counterpart in roots. Suggested by Inouye et al. (1970); Inouye (1971), gentiopicroside is generated from sweroside via swertiamarin in *G. triflora*. As a result of successively chemical conversion in one pathway, it was acceptable that the enhancement of loganic aicd's content would increase the amount of swertiamarin and sweroside, whose levels induced the gentiopicroside's content to elevate. Gentiopicroside generated obviously positive correlations (*p* < 0.01) with sweroside in leaves and swertiamarin in roots. The flower produced adverse results that sweroside showed a remarkably inverse correlation with swertiamarin in this tissue (*p* < 0.01) and gentiopicroside in roots (*p* < 0.01). What's more, the positive correlation (*p* < 0.05) was found between sweroside in stems and roots. However, while a large amount of gene related to the pathway express in leaves, there was not striking connection between gene expression profile and accumulation of (*seco*-) iridoid glycosides in Gentianaceae plants (Courdavault et al., 2014; Luca et al., 2014; Liu Y. et al., 2017). Thus, information obtained from different organs in present work might reflect the transporting relationship in inter-tissues.

**Table 2:** Pearson's correlation coefficients for contents of gentiopicroside, loganic acid, sweroside, and swertiamarin in samples collected from Dali.

**Compounds**	**Flower**				**Leave**				**Root**				**Stem**			
	**LA**	**ST**	**GE**	**SO**	**LA**	**ST**	**GE**	**SO**	**LA**	**ST**	**GE**	**SO**	**LA**	**ST**	**GE**	**SO**
**Flower**																
LA	1.00															
ST	0.34	1.00														
GE	0.24	0.42	1.00													
SO	−0.36	−0.76^*^	0.03	1.00												
**Leave**																
LA	−0.49	−0.61	−0.52	0.22	1.00											
ST	0.27	0.44	−0.12	−0.63	−0.34	1.00										
GE	−0.19	−0.3	−0.52	−0.20	0.80	0.24	1.00									
SO	−0.34	−0.59	−0.37	0.27	0.87^**^	−0.17	0.81^**^	1.00								
**Root**																
LA	−0.34	−0.47	−0.66	0.07	0.78	−0.40	0.54	0.54	1.00							
ST	0.28	0.21	−0.25	−0.40	−0.44	0.41	−0.36	−0.50	−0.08	1.00						
GE	0.28	0.09	−0.39	−0.38	−0.26	0.49	−0.10	−0.20	0.15	0.86^**^	1.00					
SO	−0.32	−0.45	−0.76^*^	0.07	0.49	−0.04	0.37	0.43	0.81^**^	0.26	0.57	1.00				
**Stem**																
LA	0.02	−0.15	0.08	0.50	−0.06	−0.39	−0.21	0.23	0.00	−0.43	−0.12	0.15	1.00			
ST	−0.35	−0.37	−0.03	0.56	0.14	−0.17	0.02	0.49	−0.07	−0.31	−0.04	0.22	0.76^*^	1.00		
GE	−0.20	−0.46	−0.59	0.46	0.36	−0.27	0.21	0.48	0.31	−0.20	0.00	0.45	0.67	0.68^*^	1.00	
SO	0.01	−0.46	−0.67^*^	0.24	0.37	−0.28	0.24	0.47	0.64	−0.09	0.30	0.77^*^	0.66^*^	0.46	0.77^**^	1.00

* p < 0.05;

** *p < 0.01*.

*LA, loganic acid; ST, swertiamarin; GE, gentiopicroside; SO, sweroside*.

The results of association analysis for samples from other two big producing areas were listed in Tables S2–S6. From the Tables S2, S3, compounds' bonds in inter-tissues accounted for around 50% of total relationships in samples from northwestern Yunnan, a low-temperature area. Connections between gentiopicroside, loganic acid, sweroside, and swertiamarin in roots and leaves were dominant in samples from Lijiang (Table S2) and Diqing (Table S3), followed by that between stems and leaves and between stems and roots. Of the broad positive correlations of target compounds in roots and leaves, the connection between loganic acid and sweroside was the most significant (*p* < 0.01) in both two areas' samples along with that between loganic acid and swertiamarin in samples from Diqing. It was true that ties between compounds in stems and leaves from Lijiang (*p* < 0.01) were solidified compared with Diqing (*p* < 0.05). The result of that sweroside in roots separately showed apparent links to gentiopicroside and sweroside (*p* < 0.01) in stems from Lijiang was agree reasonably well with that of Diqing. Differently, the core relationship of central Yunnan's samples (Tables S4–S6) was the correlation between componuds in inter-tissues, which was completely on the opposing side of western Yunnan's materials. Tissues where the compounds' bonds happened in were slightly different despite the three origins belonging to central Yunnan, a high-humidity area, finding them on the same inter-tissues relationship. The significantly positive correlation (*p* < 0.01) between loganic acids in stems and roots was found in samples from Qujing (Table S4), whereas that between swertiamarins in stems and leaves was observed in samples from Yuxi (Table S5). For samples from Kunming (Table S6), loganic acids in flowers correlated positively (*p* < 0.01) to sweroside and swertiamarin in roots, respectively. These results revealed that chemical variation in *G. rigescens* showed strongly detectable associated with environmental conditions impacting the accumulation of iridoid glycosides by adjusting the transformation of intermediates but also the transport of them between different tissues.

### Principal component analysis

**Figure 3:**
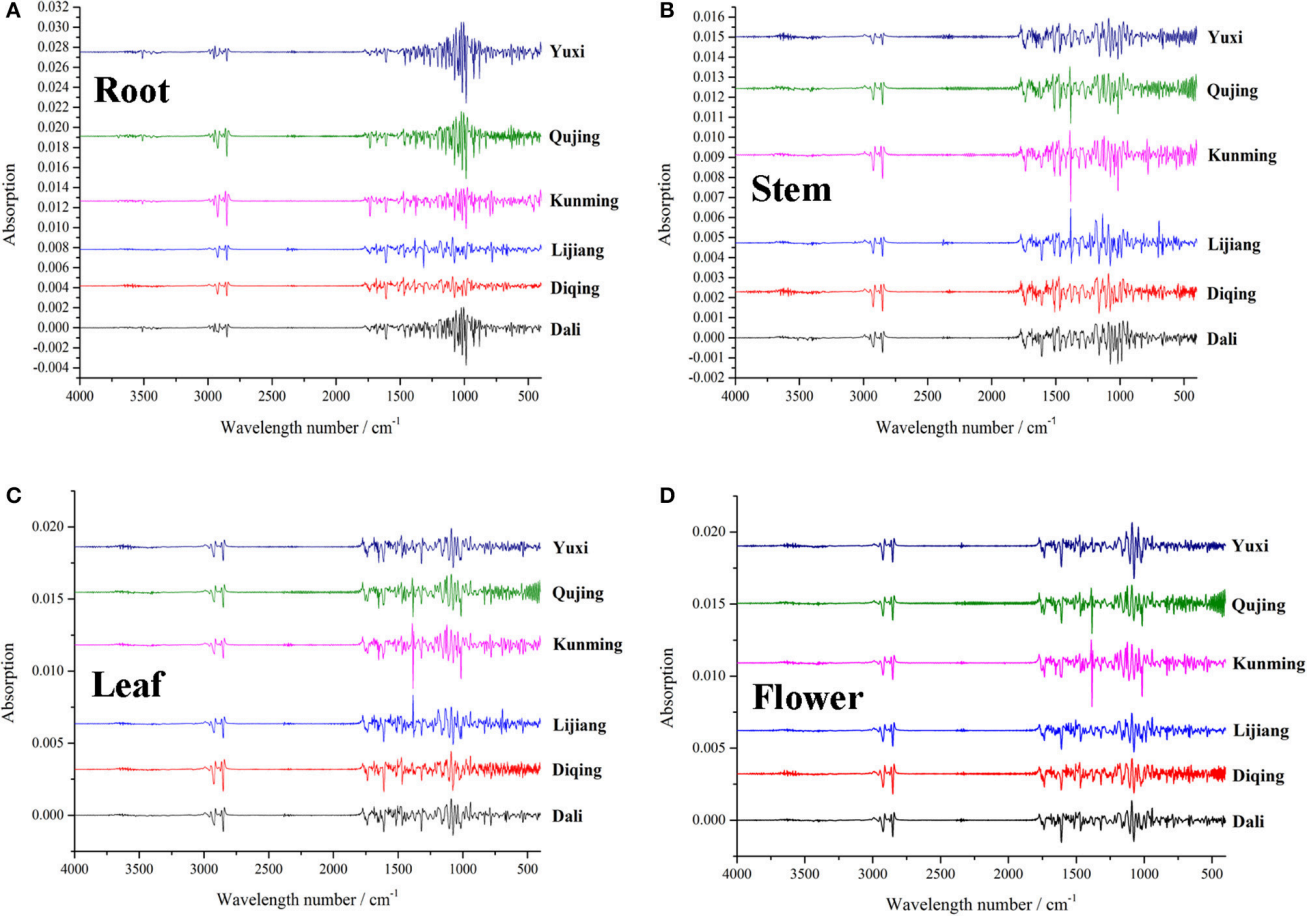
Second derivative spectral details of root **(A)**, stem **(B)**, leaf **(C)** and flower **(D)** samples of *G. rigescens* from six geographic origins.

For a long time, GR materials collected from different places by cultivators or farmers have been sold to customers and patients in markets, whose different pharmacological activities have been proven by quiet a number of clinical experiences for centuries. The important goal of quality control of GR is to gain insight into the comprehensive information of secondary metabolites in GR, which can be accomplished by the application of FTIR that provides global messages into the overall secondary metabolites through an easy and rapid process (Surewicz et al., 1993). In this work, IR data set subjected to second derivative were loaded into R language for PCA, a non-supervision algorithm concerned with finding classes of similar objects by reducing dimensionality of the data set while retaining most of variation (Wold et al., 1987; Ringnér, 2008). In our previous paper (Wu et al., 2017), classification prediction models were established using PLS-DA, a method required the allocation of observation to sets of a priori defined classes, which generated a confusion that the classification result is due to the chemistry properties or the priori definition (Härdle et al., 2017). The artificial factor could be eliminated through the PCA method, which makes the classification more objective compared to the PLS-DA. Samples second derivative spectra of IR from each geographic origin are given in Figure 3.

**Figure 4:**
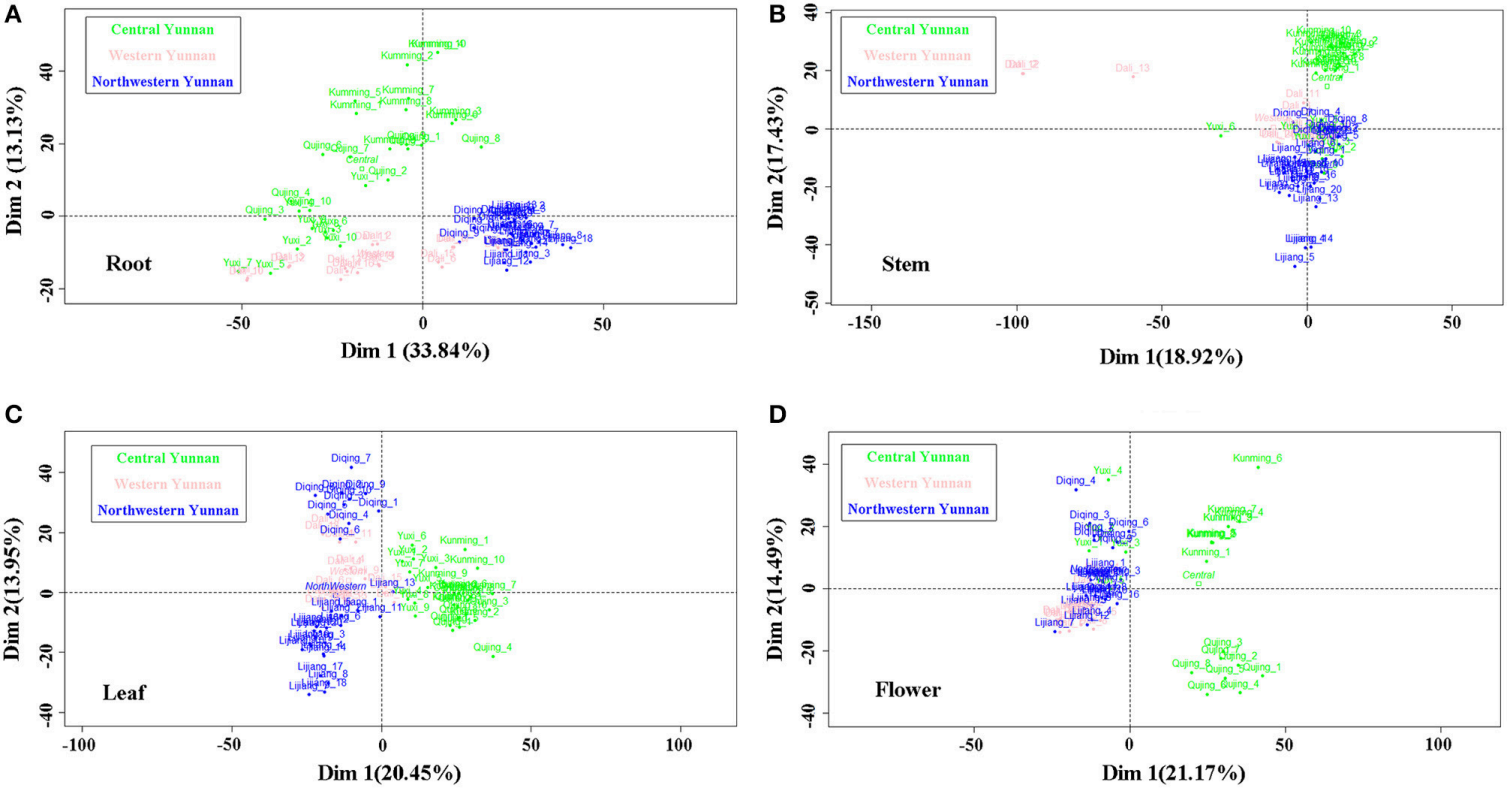
Scores plot for the first and second dimensions for root **(A)**, stem **(B)**, leaf **(C)** and flower **(D)** samples of *G. rigescens* from three large producing areas i.e. Central Yunnan, Western Yunnan and Northwestern Yunnan.

**Figure 5:**
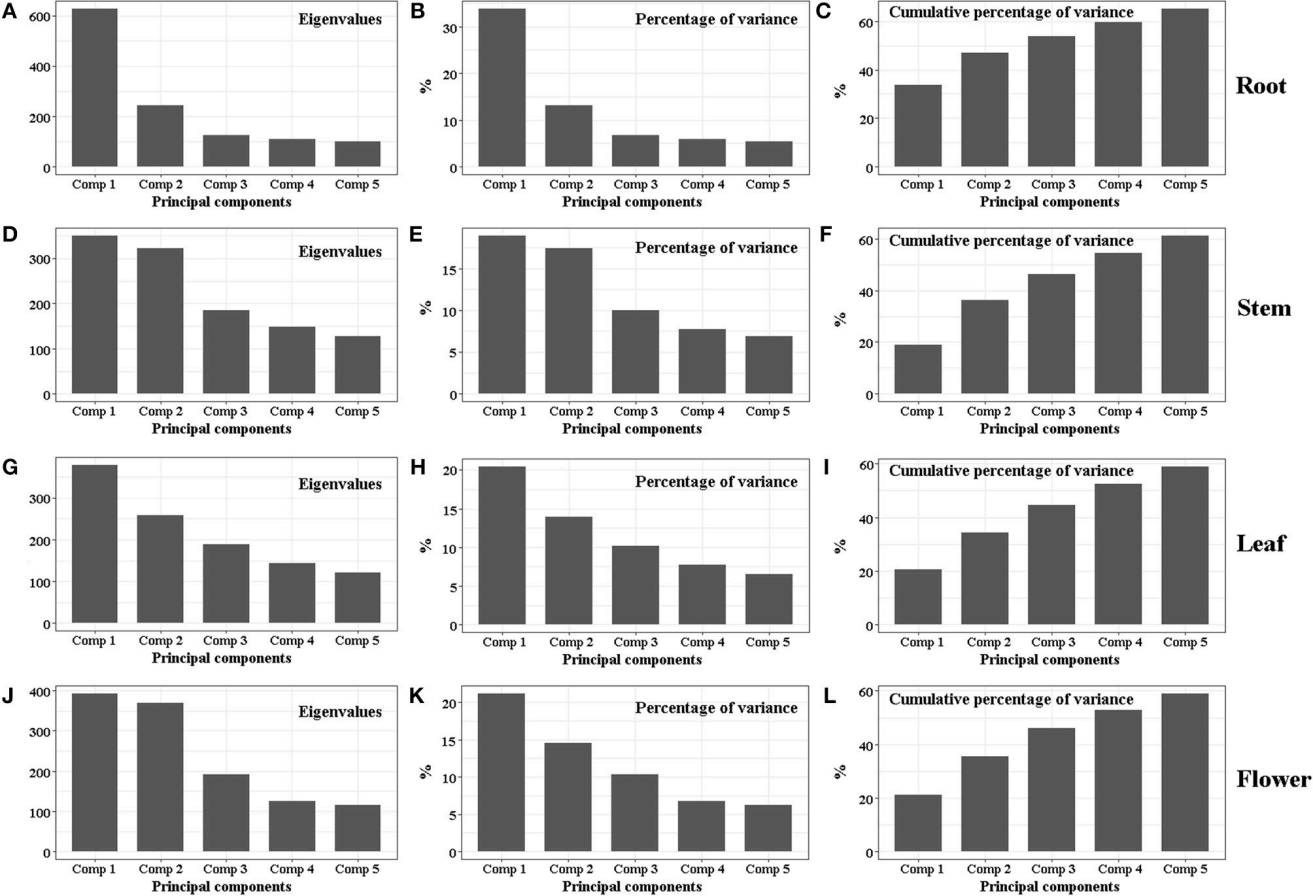
Eigenvalue, percentage of variance and cumulative percentage of variance of PCA for root **(A–C)**, stem **(D–F)**, leaf **(G–I)** and flower **(J–L)** samples collected from three large producing areas including six geographic origins.

The classification data from the 240 samples of different tissues derived from six geographic origins are displayed as scores plots of two dominant dimensions given in Figure 4. Parameters including percentages of two dominant dimensions in total variability, cumulative percentage of variance, eigenvalues were showed in Figure 5. The Figure 4A reveals root samples from northwestern Yunnan can be separated well with the Dim 1. The Dim 2, in turn, can separate samples from central Yunnan well; but there was poor aggregation between Kunming samples and Qujing and Yuxi samples which completely overlapped. Stem samples from central Yunan were recognized very well with the Dim 2 except for Yuxi ones, according to Figure 4B. Instead, there exists the poor separation among Dali (western Yunnan), Diqing (northwestern Yunnan) and Yuxi (central Yunnan) samples. Despite long distance between leaf samples from Lijiang and Diqing, the good discriminant ability for the three large producing areas was clearly observed (Figure 4C). For flowers, samples from central Yunnan were separated well with the Dim 1, except those from Yuxi (Figure 4D). The Dim 2 allowed for good separation for Diqing and Lijiang samples and Kunming and Qujing samples, reflecting the inter-class variability in the Dim 2.

From the analytical data set of IR, 52 important variables i.e., wavelength numbers were filtered and assigned by the cos2 method (Figure S3) and previous literatures, respectively. Cos2 values for these wavelength numbers are high, indicating large discriminatory ability in the classification. Unabridged lists of main peak assignments are given in Table S7. The characteristics in root samples from northwestern Yunnan were regions at 620–630, 890, 920–1,160, 1,200–1,210, 2,970, and 3,490–3,520 cm^−1^. Many of these were consistent with carbohydrates, phenols, benzoates, terpenoids, and xanthones (Kizil et al., 2002; Ivanova and Singh, 2003; Xu et al., 2007; Gao et al., 2010; Zhao et al., 2015; Miskolczi et al., 2003). Reporting in the paper that analyzed chemical compounds of three Gentianaceae species, Aberham et al. (2011) suggested that absorption bands at 3,413, 2,935, 1,208, 1,147, 923, and 627 cm^−1^ indicated the existence of xanthones. The root samples from central Yunan specially contained absorption regions at 620–630, 920–990, 2,970, and 3,490–3,520 cm^−1^, demonstrating existences of carbohydrates, benzoates and triterpenoids. Stem samples from central Yunnan had unique bands at 890, 920–930, 970–1,070, and 1,160 cm^−1^, which attributed to saccharides and aliphatic alcohols (Cael et al., 1975; Jouraiphy et al., 2008; Shen et al., 2016). The frequencies at 560–620, 670–710, 1,160–1,190, and 1,367 cm^−1^ were classified as important variables for the leaf samples from central Yunnan. They were indicative of phosphate, aromatic hydrocarbons, polyols, and celluloses (Hren et al., 2000; Garside and Wyeth, 2003; Chandran et al., 2006; Gerçel et al., 2007). The same important variables were observed for leaf samples from northwestern Yunnan, although no overlapping was found in samples from Lijiang and Diqing. Flower samples from central Yunnan could be distinguished by aliphatic alcohols, saccharides and terpenes (Jouraiphy et al., 2008; Qi et al., 2017b), namely, the bands at 1,010–1,110 and 1,200–1,210 cm^−1^. Characteristic compounds of flower samples from Diqing and Lijiang and Kunming and Qujing were large amount of saccharides, silicates, sulfates and terpenes (Zhbankov et al., 1997; Tatzber et al., 2007; Wang et al., 2011), i.e., the bands at 622, 1,016, 1,180–1,190, and 1,210 cm^−1^. The absorption band at 2,000–2,550 cm^−1^ attributed to the CO_2_ originated from air was removed. Elucidating these facts in conjunction with the habitat information (Table 1 and Figure S1), the observed classification results can be easily rationalized in terms of environmental conditions. The difference on chemical compounds between Diqing and Lijiang was likely influenced by geographic factors, especially the elevation.

Particularly problematic was the reorganization by scores values of samples from Kunming and Qujing, which grew with pine trees and weeds, respectively. In order to understand what makes poor aggregation between these samples, the PCA was performed using second derivatives of IR data set of samples lived in fir woods, pine woods and weeds. Figure S4 of the Supplemental files gives parameters to demonstrate the robustness of PCA. The classification data from the 472 samples stemmed from different habitats including fir woods, pine woods, and weeds were showed as scores plot of the two dominant dimensions given in Figure S5. That samples were divided into three groups along the habitats was not found even overlapped completely, regardless of tissues. Therefore, the likelihood that associated plants influenced chemical constituents in GR was extremely low, which was adverse with the result of study by Chu et al. (2016). We emphasize here that this result has been tentatively discovered through samples collected from natural setting. Single factor i.e., different associated plants experiments combined with agronomy, phytophysiology and molecular biology is needed. With strong supports from these facts, geographic factors could be credited as playing a big part in chemical constituents. On the other hand, roots, stems, leaves and flowers of GR displayed different responses to the environment.

### Method validation

The external standard method was employed to calculate the contents of four compounds in *G. rigescens*. The calibration curves of four compounds showed good linearity (*R*^2^ ≥ 0.99) in all cases. The LODs and LOQs were defined in the range 2.38–48.69 μg/mL and 8.06–158.70 μg/mL, respectively. The relative standard deviation (RSD%) for the peak area (*P*_*a*_) and retention time (*R*_*t*_) were below 2.90% for the intraday experiment and 3.80% for the interday experiment. The analytes demonstrated acceptable recovery efficiency (96–106%). The result was listed in Table 3.

**Table 3:** Result of the validation of the target quantitative method.

**Compound**	**LOD (μg/mL)**	**LOQ (μg/mL)**	**Intraday precision RSD, (%) (*****n***** = 3)**		**Interday precision RSD (%) (*****n***** = 3×3)**		**Equation**	***R***^2^	**Recovery (%)**
			***R_*t*_***	***P_*a*_***	***R_*t*_***	***P_*a*_***			
Gentiopicroside	48.7	158.7	0.08	0.54	0.28	0.97	y = 5767x+65	0.99	106.3
Loganic acid	5.7	19.3	0.74	0.96	3.7	1.9	y = 5514x+33	0.99	98.4
Swertiamarin	2.4	8.1	1.1	1.4	0.65	1.4	y = 4243x−15	0.99	98.5
Sweroside	3.1	11.3	2.9	0.31	2.6	0.29	y = 6948x +42	0.99	96.3

*R_t_: Retention time; P_a_: Peak aera*.

## Conclusion

The HPLC-DAD combined with FTIR analysis of different tissues of *G. rigescens* collected from six geographic origins further provided information on content of four iridoid glycosides and function group of other compounds. Gentiopicroside was the major compound in GR. The content of iridoid glycoside and chemical profile were highly depended on the climate and geography of the growth sites, resulting in the fluctuation of medicinal quality. The present work provided global information on the chemical profile and content of major iridoid glycosides in *G. rigescens* originated from six different origins. Temperature, water and altitude factors are worth valuing in the cultivation, a useful tool for controlling quality of herbal medicines systematically.

## Author contributions

H-YH and Y-ZW designed the project, processed plant collection. JL processed literature review, experimental work, data collection and analysis and manuscript drafting. JZ and Y-LZ helped in statistical analysis and manuscript revision. All authors read and approved the final manuscript for publication.

### Conflict of interest statement

The authors declare that the research was conducted in the absence of any commercial or financial relationships that could be construed as a potential conflict of interest.

## Supplementary material

Figure S1 Annual mean precipitation **(A)**, annual mean relative humidity **(B)** and annual mean temperature **(C)** data of six geographic origins of *G. rigescens* downloading from Climatic Data Center, National Meteorological Information Center, China Meteorological Administration.

Figure S2 The total contents of gentiopicroside **(A)**, loganic acid **(B)**, sweroside **(C)** and sweritiamarin **(D)** in whole plant samples from three large producing areas composed of six geographic origins (mg/g, *n* = 10).

Figure S3 Variable plots of PCA for the FTIR wavelength number of samples collected from three big producing areas including six geographic origins.

Figure S4 Scores plot for the first and second dimensions for the samples living in fir woods, pine woods and weeds.

Figure S5 Eigenvalue, percentage of variance and cumulative percentage of variance of PCA for samples living in fir woods, pine woods and weeds.

Table S1 The contents of chemical compositions (mg/g raw materials ± SE) in four iridoid glycosdies in samples from six geographic origins.

Table S2 Pearson's correlation coefficients for contents of gentiopicroside, loganic acid, sweroside, and swertiamarin in samples collected from Lijiang.

Table S3 Pearson's correlation coefficients for contents of gentiopicroside, loganic acid, sweroside, and swertiamarin in samples collected from Diqing.

Table S4 Pearson's correlation coefficients for contents of gentiopicroside, loganic acid, sweroside, and swertiamarin in samples collected from Qujing.

Table S5 Pearson's correlation coefficients for contents of gentiopicroside, loganic acid, sweroside, and swertiamarin in samples collected from Yuxi.

Table S6 Pearson's correlation coefficients for contents of gentiopicroside, loganic acid, sweroside, and swertiamarin in samples collected from Kuming.

Table S7 Fourier transformed infrared (FTIR) wavelength numbers, in cm-1, and their respective tentative assignments.
