# Supplementary figures and images for: Comprehensive Quality Assessment Based Specific Chemical Profiles for Geographic and Tissue Variation in Gentiana rigescens Using HPLC and FTIR Method Combined with Principal Component Analysis
Source: Front Chem. 2017 Dec 22;5:125. doi: 10.3389/fchem.2017.00125 (PMC5743669; doi:10.3389/fchem.2017.00125)

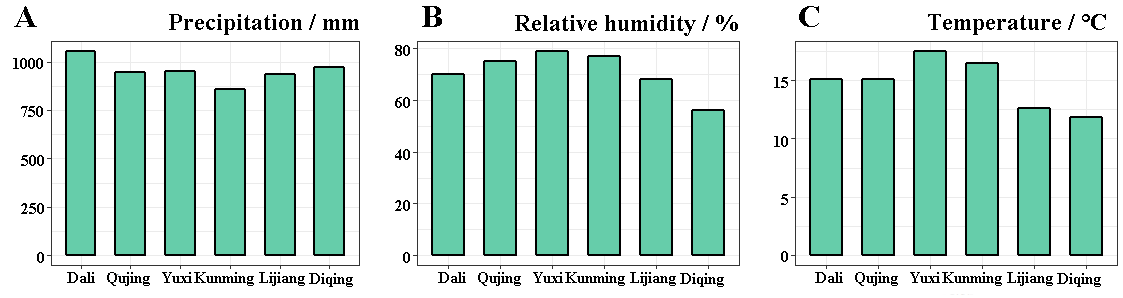

Supplement: Figure S1 — Annual mean precipitation (A), annual mean relative humidity (B) and annual mean temperature (C) data of six geographic origins of G. rigescens downloading from Climatic Data Center, National Meteorological Information Center, China Meteorological Administration. [file Image1.tif]

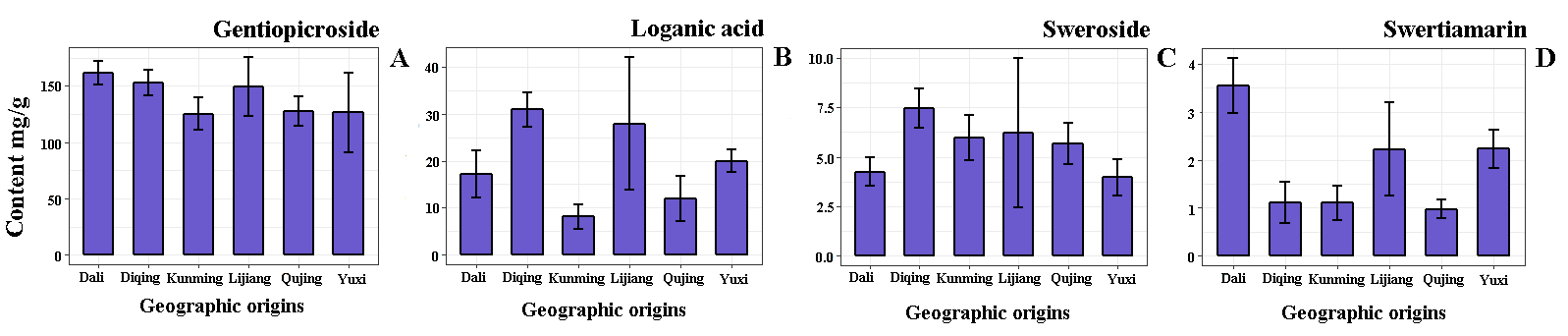

Supplement: Figure S2 — The total contents of gentiopicroside (A), loganic acid (B), sweroside (C) and sweritiamarin (D) in whole plant samples from three large producing areas composed of six geographic origins (mg/g, n = 10). [file Image2.TIF]

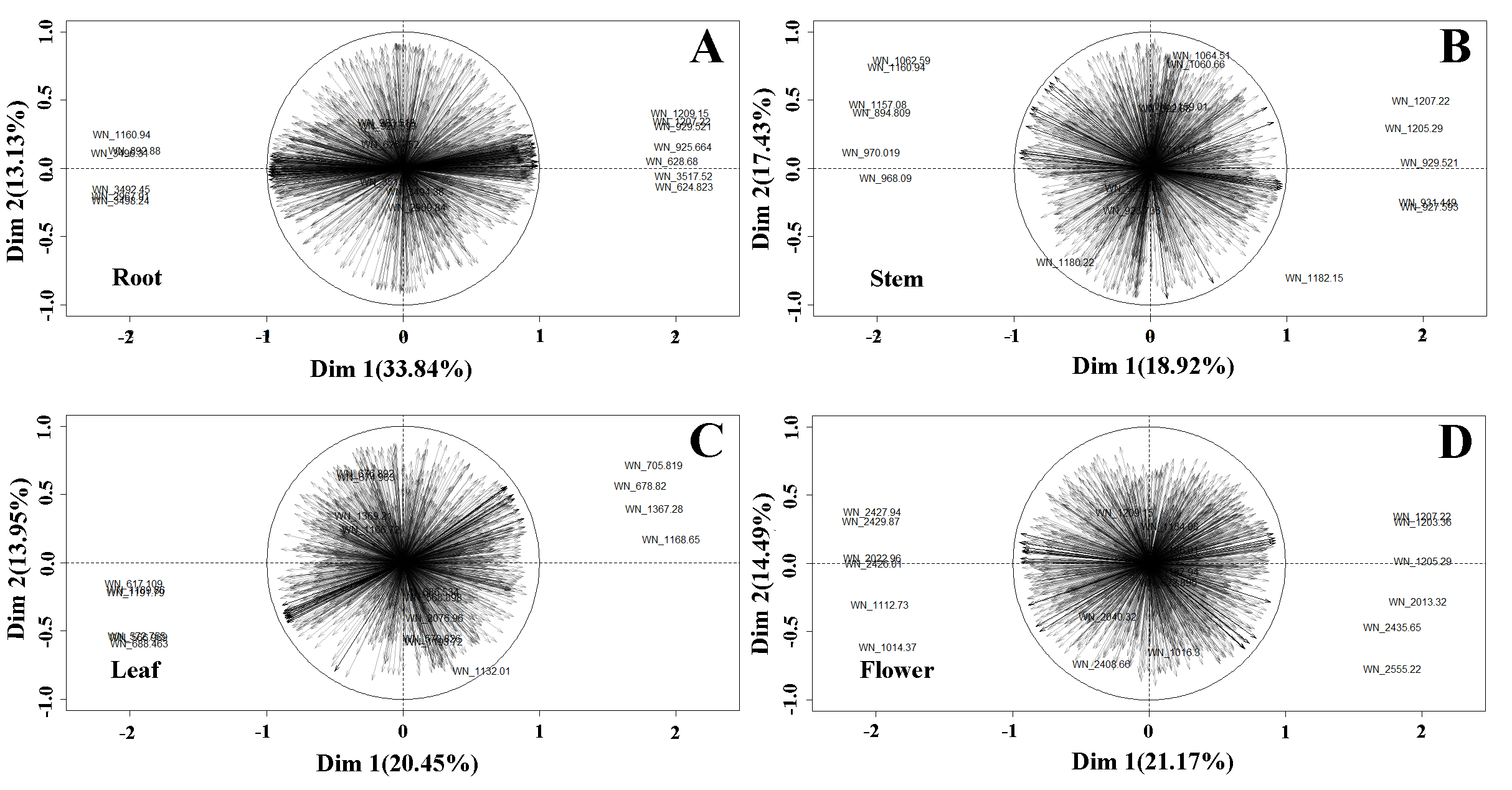

Supplement: Figure S3 — Variable plots of PCA for the FTIR wavelength number of samples collected from three big producing areas including six geographic origins. [file Image3.TIF]

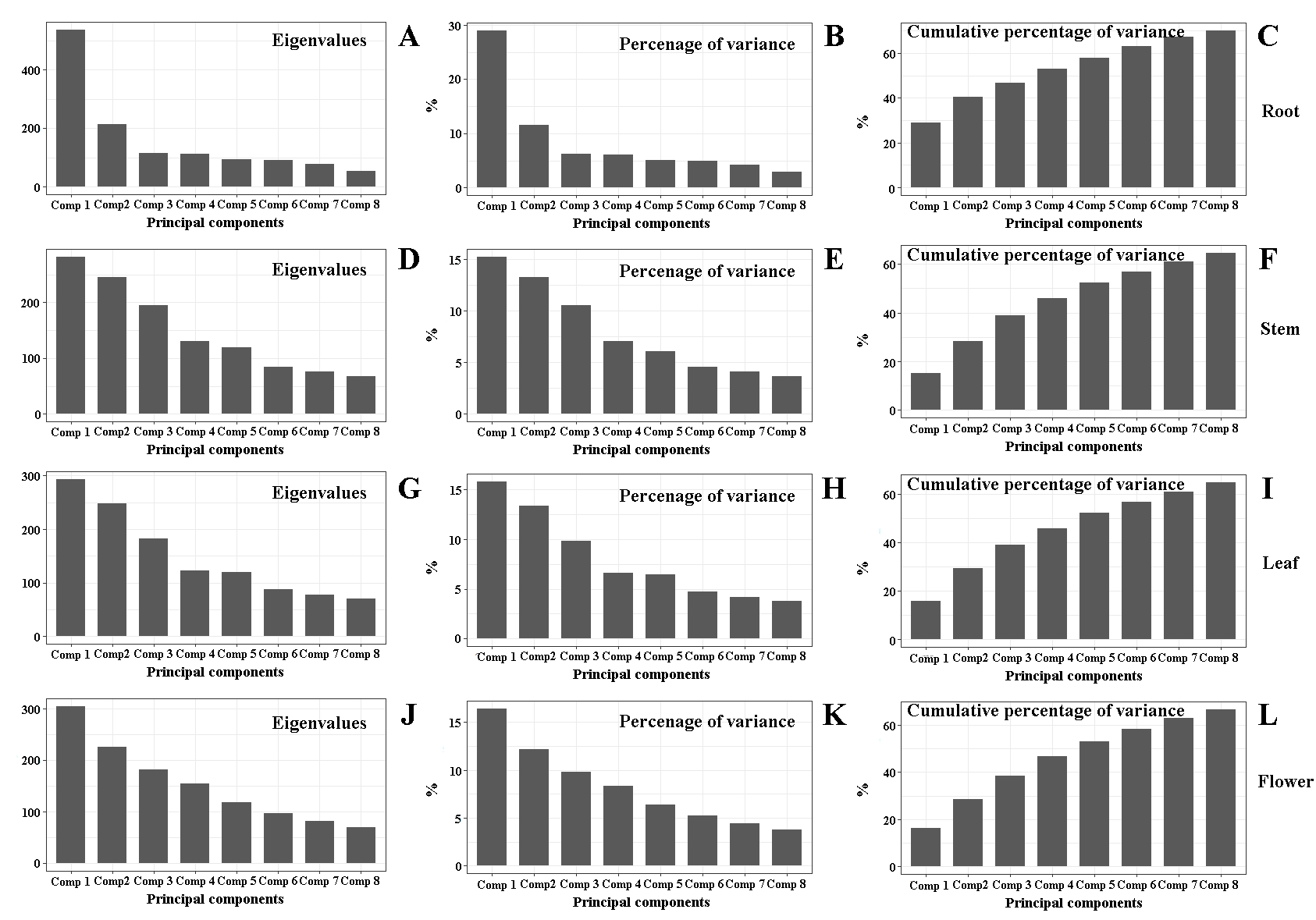

Supplement: Figure S4 — Scores plot for the first and second dimensions for the samples living in fir woods, pine woods and weeds. [file Image4.TIF]

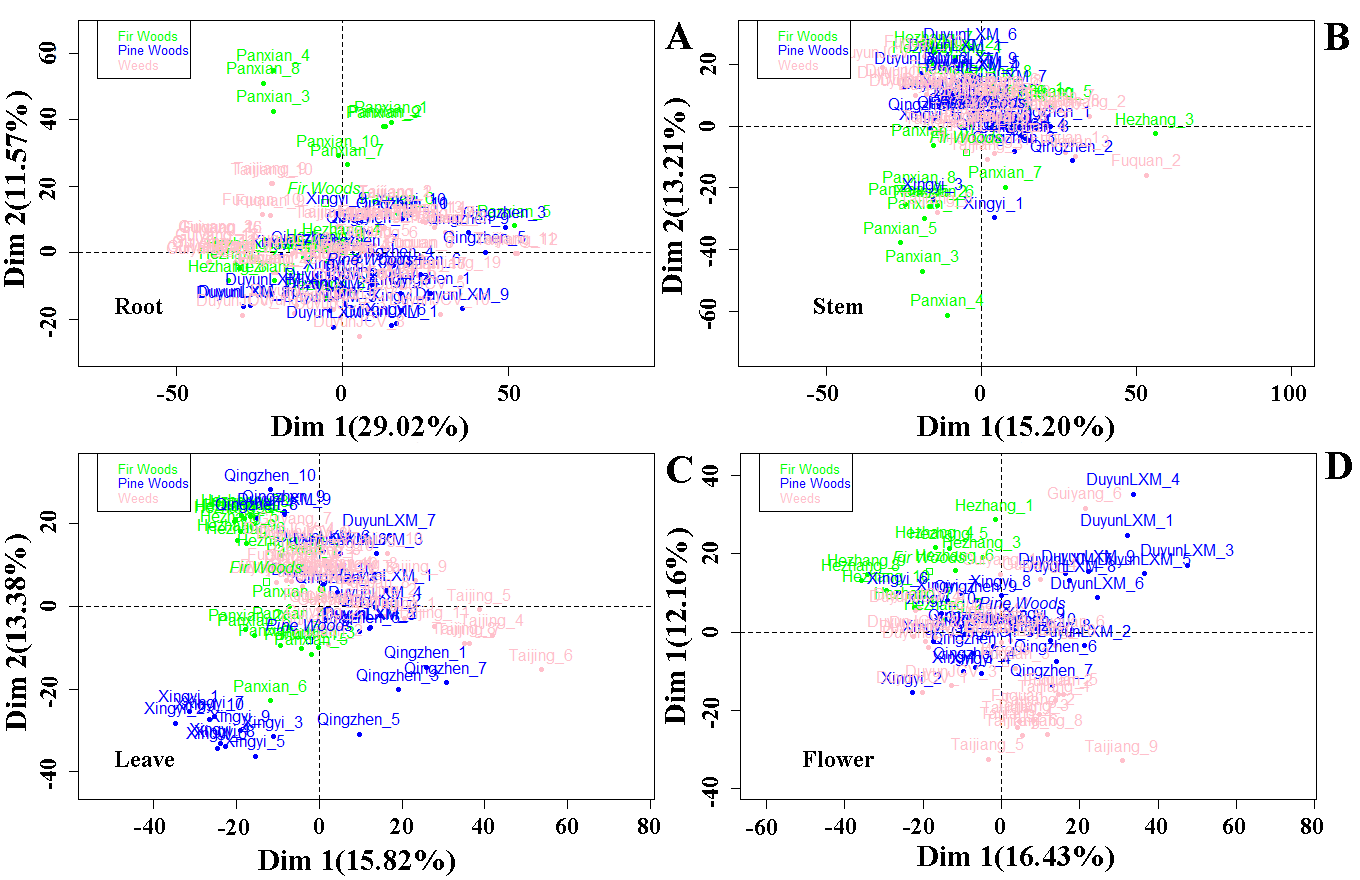

Supplement: Figure S5 — Eigenvalue, percentage of variance and cumulative percentage of variance of PCA for samples living in fir woods, pine woods and weeds. [file Image5.TIF]
