# Supplementary material for: Comprehensive Quality Assessment Based Specific Chemical Profiles for Geographic and Tissue Variation in Gentiana rigescens Using HPLC and FTIR Method Combined with Principal Component Analysis
Source: Front Chem. 2017 Dec 22;5:125. doi: 10.3389/fchem.2017.00125 (PMC5743669; doi:10.3389/fchem.2017.00125)
Supplement: Table S1 — The contents of chemical compositions (mg/g raw materials ± SE) in four iridoid glycosdies in samples from six geographic origins. [file Table1.DOCX]

**Table S1** The contents of chemical compositions (mg/g raw materials ± SE) in four iridoid glycosdies in samples from six geographic origins

| **Samples** | **Gentiopicroside** | **Loganic acid** | **Sweroside** | **Swertiamarin** |
| --- | --- | --- | --- | --- |
| **Flower** |  |  |  |  |
| Dali | 47.78±2.04^b^ | 2.90±0.52^ab^ | 1.66±0.09^ac^ | 0.52±0.10^a^ |
| Yuxi | 41.87±7.26^ab^ | 5.28±1.04^bc^ | 1.78±0.46^ac^ | 0.38±0.12^a^ |
| Kunming | 38.47±4.22^ab^ | 1.70±0.43^a^ | 2.22±0.22^bc^ | 0.20±0.02^a^ |
| Qujing | 42.87±2.52^ab^ | 2.91±0.60^ac^ | 2.52±0.24^c^ | 0.16±0.04^a^ |
| Lijiang | 41.30±1.25^ab^ | 5.41±1.28^bc^ | 1.36±0.06^ab^ | 0.19±0.06^a^ |
| Diqing | 29.66±2.11^a^ | 5.73±0.43^c^ | 1.75±0.06^ac^ | 0.11±0.04^a^ |
| **Leave** |  |  |  |  |
| Dali | 27.77±2.92^bc^ | 1.41±0.49^a^ | 0.95±0.09^ab^ | 1.78±0.15^d^ |
| Yuxi | 24.68±3.76^ac^ | 2.68±0.45^a^ | 0.88±0.19^ab^ | 0.97±0.21^bc^ |
| Kunming | 24.73±2.97^ac^ | 1.59±1.18^a^ | 1.65±0.14^b^ | 0.18±0.04^a^ |
| Qujing | 31.35±3.44^c^ | 0.66±0.25^a^ | 1.42±0.18^b^ | 0.43±0.04^ac^ |
| Lijiang | 35.61±3.61^c^ | 3.77±1.47^a^ | 1.44±0.15^b^ | 1.10±0.16^cd^ |
| Diqing | 52.33±3.57^d^ | 14.61±1.26^b^ | 3.09±0.33^c^ | 0.39±0.06^ab^ |
| **Root** |  |  |  |  |
| Dali | 53.40±2.34^a^ | 9.39±1.15^ab^ | 0.96±0.16^a^ | 0.83±0.07^b^ |
| Yuxi | 43.73±1.47^a^ | 10.46±1.03^b^ | 1.07±0.08^a^ | 0.89±0.05^b^ |
| Kunming | 44.84±2.46^a^ | 5.24±0.71^a^ | 1.23±0.17^a^ | 0.70±0.11^ab^ |
| Qujing | 39.41±2.24^a^ | 7.05±1.11^ab^ | 1.07±0.07^a^ | 0.32±0.02^a^ |
| Lijiang | 40.91±5.05^a^ | 11.13±1.23^b^ | 2.07±0.95^ab^ | 0.59±0.16^ab^ |
| Diqing | 51.97±6.13^a^ | 6.61±0.81^ab^ | 1.64±0.18^a^ | 0.63±0.14^ab^ |
| **Stem** |  |  |  |  |
| Dali | 32.49±3.57^c^ | 3.99±1.54^a^ | 0.70±0.07^ac^ | 0.39±0.07^c^ |
| Yuxi | 15.87±2.32^ab^ | 2.63±0.75^a^ | 0.54±0.05^ab^ | 0.21±0.06^ac^ |
| Kunming | 19.50±1.27^ab^ | 1.42±0.31^a^ | 0.77±0.08^ac^ | 0.00±0.00^a^ |
| Qujing | 14.55±0.75^ab^ | 1.42±0.36^a^ | 0.54±0.03^ab^ | 0±0.00^a^ |
| Lijiang | 26.61±4.14^bc^ | 2.83±0.91^a^ | 1.12±0.27^c^ | 0.21±0.08^ac^ |
| Diqing | 18.11±5.45^ab^ | 4.05±0.96^a^ | 1.01±0.11^bc^ | 0.01±0.06^ab^ |
| **Total** |  |  |  |  |
| Dali | 161.80±4.52^b^ | 17.16±2.16^b^ | 4.24±0.31^a^ | 3.55±0.25^c^ |
| Yuxi | 126.41±13.64^a^ | 19.96±0.92^bc^ | 3.96±0.35^a^ | 2.22±0.15^ab^ |
| Kunming | 125.31±6.09^a^ | 8.18±1.11^a^ | 5.95±0.48^ab^ | 1.10±0.14^a^ |
| Qujing | 127.62±5.59^a^ | 11.97±2.07^ab^ | 5.69±0.44^ab^ | 0.98±0.06^a^ |
| Lijiang | 149.65±10.70^ab^ | 27.91±4.45^cd^ | 6.23±1.60^ab^ | 2.22±0.40^b^ |
| Diqing | 153.17±4.89^ab^ | 30.88±1.56^d^ | 7.47±0.43^b^ | 1.11±0.18^a^ |

Each value is expressed as mean ± SE (n = 10)

Values followed by same letters are not significantly (*p* > 0.05) different in Turkey’s test
