# Supplementary material for: Comprehensive Quality Assessment Based Specific Chemical Profiles for Geographic and Tissue Variation in Gentiana rigescens Using HPLC and FTIR Method Combined with Principal Component Analysis
Source: Front Chem. 2017 Dec 22;5:125. doi: 10.3389/fchem.2017.00125 (PMC5743669; doi:10.3389/fchem.2017.00125)
Supplement: Table S2 — Pearson's correlation coefficients for contents of gentiopicroside, loganic acid, sweroside, and swertiamarin in samples collected from Lijiang. [file Table2.DOCX]

**Table S2** Pearson’s correlation coefficients for contents of gentiopicroside, loganic acid, sweroside and swertiamarin in samples collected from Lijiang.

| **Compounds** | **Flower** | | | | **Leave** | | | | **Root** | | | | **Stem** | | | |
| --- | --- | --- | --- | --- | --- | --- | --- | --- | --- | --- | --- | --- | --- | --- | --- | --- |
|  | LA | ST | GE | SO | LA | ST | GE | SO | LA | ST | GE | SO | LA | ST | GE | SO |
| **Flower** |  |  |  |  |  |  |  |  |  |  |  |  |  |  |  |  |
| LA | 1.00 |  |  |  |  |  |  |  |  |  |  |  |  |  |  |  |
| ST | 0.71 | 1.00 |  |  |  |  |  |  |  |  |  |  |  |  |  |  |
| GE | -0.28 | -0.02 | 1.00 |  |  |  |  |  |  |  |  |  |  |  |  |  |
| SO | -0.11 | -0.22 | 0.31 | 1.00 |  |  |  |  |  |  |  |  |  |  |  |  |
| **Leave** |  |  |  |  |  |  |  |  |  |  |  |  |  |  |  |  |
| LA | -0.29 | -0.43 | -0.12 | -0.73* | 1.00 |  |  |  |  |  |  |  |  |  |  |  |
| ST | -0.08 | -0.23 | -0.19 | -0.50 | 0.60 | 1.00 |  |  |  |  |  |  |  |  |  |  |
| GE | -0.47 | -0.60 | 0.05 | -0.56 | 0.84 | 0.68* | 1.00 |  |  |  |  |  |  |  |  |  |
| SO | -0.63 | -0.62 | 0.00 | -0.44 | 0.57 | 0.63 | 0.76* | 1.00 |  |  |  |  |  |  |  |  |
| **Root** |  |  |  |  |  |  |  |  |  |  |  |  |  |  |  |  |
| LA | -0.06 | -0.25 | -0.41 | -0.55 | 0.76 | 0.79* | 0.55 | 0.48 | 1.00 |  |  |  |  |  |  |  |
| ST | 0.01 | -0.40 | -0.45 | -0.65 | 0.85 | 0.74* | 0.67 | 0.59 | 0.84** | 1.00 |  |  |  |  |  |  |
| GE | -0.30 | 0.30 | -0.18 | -0.43 | 0.03 | 0.16 | -0.01 | 0.38 | 0.06 | -0.02 | 1.00 |  |  |  |  |  |
| SO | -0.32 | -0.45 | -0.23 | -0.69 | 0.96** | 0.67* | 0.77** | 0.63 | 0.82** | 0.90** | 0.19 | 1.00 |  |  |  |  |
| **Stem** |  |  |  |  |  |  |  |  |  |  |  |  |  |  |  |  |
| LA | 0.93 | 0.76 | -0.84 | 0.41 | -0.77 | -0.94* | -0.97* | -1.00* | -0.85 | -0.72 | -0.23 | -0.84 | 1.00 |  |  |  |
| ST | 0.42 | 0.48 | 0.43 | -0.01 | 0.00 | 0.14 | -0.25 | -0.30 | 0.21 | -0.03 | -0.16 | -0.03 | -0.43 | 1.00 |  |  |
| GE | 0.02 | -0.09 | 0.06 | -0.66 | 0.79** | 0.57 | 0.51 | 0.38 | 0.64 | 0.75* | 0.16 | 0.83** | -0.73 | 0.41 | 1.00 |  |
| SO | -0.29 | -0.41 | 0.05 | -0.53 | 0.85** | 0.51 | 0.59 | 0.52 | 0.65 | 0.76* | 0.14 | 0.91** | -0.80 | 0.19 | 0.93** | 1.00 |

*: p < 0.05; **: p < 0.01
