# Supplementary material for: Comprehensive Quality Assessment Based Specific Chemical Profiles for Geographic and Tissue Variation in Gentiana rigescens Using HPLC and FTIR Method Combined with Principal Component Analysis
Source: Front Chem. 2017 Dec 22;5:125. doi: 10.3389/fchem.2017.00125 (PMC5743669; doi:10.3389/fchem.2017.00125)
Supplement: Table S4 — Pearson's correlation coefficients for contents of gentiopicroside, loganic acid, sweroside, and swertiamarin in samples collected from Qujing. [file Table4.DOCX]

**Table S4** Pearson’s correlation coefficients for contents of gentiopicroside, loganic acid, sweroside and swertiamarin in samples collected from Qujing

| **Compounds** | **Flower** | | | | **Leave** | | | | **Root** | | | | **Stem** | | | |
| --- | --- | --- | --- | --- | --- | --- | --- | --- | --- | --- | --- | --- | --- | --- | --- | --- |
|  | LA | ST | GE | SO | LA | ST | GE | SO | LA | ST | GE | SO | LA | ST | GE | SO |
| **Flower** |  |  |  |  |  |  |  |  |  |  |  |  |  |  |  |  |
| LA | 1.00 |  |  |  |  |  |  |  |  |  |  |  |  |  |  |  |
| ST | 0.24 | 1.00 |  |  |  |  |  |  |  |  |  |  |  |  |  |  |
| GE | 0.10 | 0.08 | 1.00 |  |  |  |  |  |  |  |  |  |  |  |  |  |
| SO | 0.16 | 0.38 | -0.55 | 1.00 |  |  |  |  |  |  |  |  |  |  |  |  |
| **Leave** |  |  |  |  |  |  |  |  |  |  |  |  |  |  |  |  |
| LA | 0.02 | 0.42 | 0.26 | 0.10 | 1.00 |  |  |  |  |  |  |  |  |  |  |  |
| ST | 0.18 | 0.01 | 0.74* | -0.78* | 0.44 | 1.00 |  |  |  |  |  |  |  |  |  |  |
| GE | -0.35 | -0.07 | -0.20 | 0.49 | 0.48 | -0.35 | 1.00 |  |  |  |  |  |  |  |  |  |
| SO | -0.29 | -0.04 | -0.06 | 0.37 | 0.83** | 0.14 | 0.74* | 1.00 |  |  |  |  |  |  |  |  |
| **Root** |  |  |  |  |  |  |  |  |  |  |  |  |  |  |  |  |
| LA | 0.42 | 0.97* | -0.03 | -0.15 | 0.51 | 0.40 | 0.03 | 0.09 | 1.00 |  |  |  |  |  |  |  |
| ST | 0.85 | 0.33 | 0.14 | 0.13 | 0.30 | 0.50 | -0.32 | 0.05 | 0.60 | 1.00 |  |  |  |  |  |  |
| GE | 0.36 | 0.42 | 0.18 | 0.25 | 0.51 | 0.27 | 0.23 | 0.30 | 0.40 | 0.40 | 1.00 |  |  |  |  |  |
| SO | -0.33 | -0.52 | -0.37 | 0.30 | 0.34 | -0.26 | 0.75 | 0.72* | -0.15 | -0.39 | -0.23 | 1.00 |  |  |  |  |
| **Stem** |  |  |  |  |  |  |  |  |  |  |  |  |  |  |  |  |
| LA | 0.54 | 0.84* | -0.44 | 0.22 | 0.38 | 0.14 | -0.12 | 0.06 | 0.84** | 0.66* | 0.29 | -0.14 | 1.00 |  |  |  |
| ST | - | - | - | - | - | - | - | - | - | - | - | - | - | 1.00 |  |  |
| GE | 0.58 | 0.45 | -0.13 | 0.08 | -0.55 | -0.20 | -0.57 | -0.68* | 0.25 | 0.40 | -0.26 | -0.58 | 0.44 | - | 1.00 |  |
| SO | -0.14 | -0.88 | -0.10 | -0.18 | 0.18 | 0.28 | 0.13 | 0.44 | -0.08 | 0.08 | -0.28 | 0.64* | -0.05 | - | -0.33 | 1.00 |

*: p < 0.05; **: p < 0.01

-: not detected using HPLC
