# Supplementary material for: Comprehensive Quality Assessment Based Specific Chemical Profiles for Geographic and Tissue Variation in Gentiana rigescens Using HPLC and FTIR Method Combined with Principal Component Analysis
Source: Front Chem. 2017 Dec 22;5:125. doi: 10.3389/fchem.2017.00125 (PMC5743669; doi:10.3389/fchem.2017.00125)
Supplement: Table S5 — Pearson's correlation coefficients for contents of gentiopicroside, loganic acid, sweroside, and swertiamarin in samples collected from Yuxi. [file Table5.DOCX]

**Table S5** Pearson’s correlation coefficients for contents of gentiopicroside, loganic acid, sweroside and swertiamarin in samples collected from Yuxi

| **Compounds** | **Flower** | | | | **Leave** | | | | **Root** | | | | **Stem** | | | |
| --- | --- | --- | --- | --- | --- | --- | --- | --- | --- | --- | --- | --- | --- | --- | --- | --- |
|  | LA | ST | GE | SO | LA | ST | GE | SO | LA | ST | GE | SO | LA | ST | GE | SO |
| **Flower** |  |  |  |  |  |  |  |  |  |  |  |  |  |  |  |  |
| LA | 1 |  |  |  |  |  |  |  |  |  |  |  |  |  |  |  |
| ST | -0.27 | 1 |  |  |  |  |  |  |  |  |  |  |  |  |  |  |
| GE | 0.84* | -0.23 | 1 |  |  |  |  |  |  |  |  |  |  |  |  |  |
| SO | 0.90* | -0.36 | 0.92** | 1 |  |  |  |  |  |  |  |  |  |  |  |  |
| **Leave** |  |  |  |  |  |  |  |  |  |  |  |  |  |  |  |  |
| LA | 0.23 | -0.23 | 0.34 | 0.27 | 1 |  |  |  |  |  |  |  |  |  |  |  |
| ST | 0.44 | -0.89* | 0.44 | 0.58 | 0.36 | 1 |  |  |  |  |  |  |  |  |  |  |
| GE | 0.32 | -0.18 | 0.66 | 0.47 | 0.49 | 0.13 | 1 |  |  |  |  |  |  |  |  |  |
| SO | -0.31 | -0.71 | -0.35 | -0.35 | 0.77* | 0.70* | 0.21 | 1 |  |  |  |  |  |  |  |  |
| **Root** |  |  |  |  |  |  |  |  |  |  |  |  |  |  |  |  |
| LA | -0.11 | 0.28 | -0.47 | -0.13 | 0.47 | 0.13 | -0.09 | 0.51 | 1 |  |  |  |  |  |  |  |
| ST | -0.16 | 0.56 | -0.48 | -0.54 | -0.24 | -0.39 | -0.12 | -0.13 | -0.13 | 1 |  |  |  |  |  |  |
| GE | -0.01 | 0.25 | -0.15 | -0.10 | 0.67* | -0.26 | 0.29 | 0.27 | 0.37 | 0.37 | 1 |  |  |  |  |  |
| SO | -0.56 | 0.55 | -0.85* | -0.74 | -0.14 | -0.03 | -0.10 | 0.18 | 0.19 | 0.67* | 0.21 | 1 |  |  |  |  |
| **Stem** |  |  |  |  |  |  |  |  |  |  |  |  |  |  |  |  |
| LA | -0.46 | -0.39 | 0.04 | -0.25 | 0.05 | 0.44 | 0.42 | 0.33 | -0.44 | 0.18 | -0.14 | 0.38 | 1 |  |  |  |
| ST | 0.34 | -0.93* | 0.35 | 0.52 | 0.01 | 0.98** | 0.11 | 0.43 | -0.18 | -0.80 | -0.49 | -0.69 | 0.20 | 1 |  |  |
| GE | -0.08 | -0.24 | 0.45 | 0.16 | 0.08 | 0.29 | 0.55 | 0.09 | -0.65* | -0.08 | -0.16 | -0.10 | 0.85** | 0.29 | 1 |  |
| SO | -0.53 | -0.84 | -0.17 | -0.35 | -0.19 | 0.49 | 0.16 | 0.23 | -0.49 | 0.23 | -0.26 | 0.34 | 0.91** | 0.77 | 0.75* | 1 |

*: p < 0.05; **: p < 0.01
