# Supplementary material for: Comprehensive Quality Assessment Based Specific Chemical Profiles for Geographic and Tissue Variation in Gentiana rigescens Using HPLC and FTIR Method Combined with Principal Component Analysis
Source: Front Chem. 2017 Dec 22;5:125. doi: 10.3389/fchem.2017.00125 (PMC5743669; doi:10.3389/fchem.2017.00125)
Supplement: Table S6 — Pearson's correlation coefficients for contents of gentiopicroside, loganic acid, sweroside, and swertiamarin in samples collected from Kuming. [file Table6.DOCX]

**Table S6** Pearson’s correlation coefficients for contents of gentiopicroside, loganic acid, sweroside and swertiamarin in samples collected from Kuming

| **Compounds** | **Flower** | | | | **Leave** | | | | **Root** | | | | **Stem** | | | |
| --- | --- | --- | --- | --- | --- | --- | --- | --- | --- | --- | --- | --- | --- | --- | --- | --- |
|  | LA | ST | GE | SO | LA | ST | GE | SO | LA | ST | GE | SO | LA | ST | GE | SO |
| **Flower** |  |  |  |  |  |  |  |  |  |  |  |  |  |  |  |  |
| LA | 1 |  |  |  |  |  |  |  |  |  |  |  |  |  |  |  |
| ST | -0.44 | 1 |  |  |  |  |  |  |  |  |  |  |  |  |  |  |
| GE | 0.30 | 0.26 | 1 |  |  |  |  |  |  |  |  |  |  |  |  |  |
| SO | 0.26 | 0.32 | 0.47 | 1 |  |  |  |  |  |  |  |  |  |  |  |  |
| **Leave** |  |  |  |  |  |  |  |  |  |  |  |  |  |  |  |  |
| LA | 0.44 | -0.06 | 0.58 | -0.03 | 1 |  |  |  |  |  |  |  |  |  |  |  |
| ST | -0.09 | -0.28 | 0.31 | -0.19 | -0.05 | 1 |  |  |  |  |  |  |  |  |  |  |
| GE | -0.29 | 0.52 | 0.42 | -0.38 | -0.03 | 0.39 | 1 |  |  |  |  |  |  |  |  |  |
| SO | 0.68 | -0.29 | 0.12 | -0.13 | -0.19 | 0.40 | -0.25 | 1 |  |  |  |  |  |  |  |  |
| **Root** |  |  |  |  |  |  |  |  |  |  |  |  |  |  |  |  |
| LA | 0.05 | 0.17 | 0.10 | 0.59 | -0.01 | -0.14 | 0.27 | -0.53 | 1 |  |  |  |  |  |  |  |
| ST | 0.84** | -0.58 | 0.03 | 0.01 | 0.20 | -0.23 | -0.39 | 0.62 | -0.13 | 1 |  |  |  |  |  |  |
| GE | 0.69 | -0.38 | -0.13 | 0.28 | -0.30 | -0.19 | -0.06 | 0.19 | 0.62* | 0.57 | 1 |  |  |  |  |  |
| SO | 0.93** | -0.61 | 0.29 | 0.13 | -0.10 | 0.18 | -0.08 | 0.59 | -0.03 | 0.86** | 0.58 | 1 |  |  |  |  |
| **Stem** |  |  |  |  |  |  |  |  |  |  |  |  |  |  |  |  |
| LA | -0.03 | -0.57 | 0.03 | 0.15 | -0.12 | 0.4 | 0.18 | -0.52 | 0.72* | -0.19 | 0.38 | 0.02 | 1 |  |  |  |
| ST | -0.27 | 0.25 | 0.32 | -0.43 | -0.12 | 0.69 | 0.51 | 0.26 | -0.50 | -0.4 | -0.56 | -0.12 | -0.18 | 1 |  |  |
| GE | -0.10 | -0.40 | 0.02 | -0.23 | -0.21 | 0.74 | -0.17 | 0.33 | -0.48 | -0.29 | -0.27 | 0.15 | 0.07 | 0.53 | 1 |  |
| SO | 0.36 | -0.44 | 0.20 | -0.31 | -0.15 | 0.69 | -0.06 | 0.7* | -0.65* | 0.29 | -0.17 | 0.52 | -0.19 | 0.64 | 0.71* | 1 |

*: p < 0.05; **: p < 0.01
