# Supplementary material for: Comprehensive Quality Assessment Based Specific Chemical Profiles for Geographic and Tissue Variation in Gentiana rigescens Using HPLC and FTIR Method Combined with Principal Component Analysis
Source: Front Chem. 2017 Dec 22;5:125. doi: 10.3389/fchem.2017.00125 (PMC5743669; doi:10.3389/fchem.2017.00125)
Supplement: Table S7 — Fourier transformed infrared (FTIR) wavelength numbers, in cm-1, and their respective tentative assignments. [file Table7.docx]

**Table S7** Fourier transformed infrared (FTIR) wavelength numbers, in cm^-1^, and their respective tentative assignments

| **Absorption bands / cm^-1^** | **Chemical groups** | **Remarks** | **References** |
| --- | --- | --- | --- |
| 622 | pyranose ring | carbohydrates | (Kizil et al., 2002; Zhbankov et al., 1997) |
| 624 | pyranose ring | carbohydrates | (Kizil et al., 2002; Zhbankov et al., 1997) |
| 626 | pyranose ring | carbohydrates | (Kizil et al., 2002; Zhbankov et al., 1997) |
| 628 | pyranose ring | carbohydrates | (Kizil et al., 2002; Zhbankov et al., 1997) |
| 674 | *δ*(C-H) | aromatic hydrocarbones | (Hren et al., 2000) |
| 676 | *δ*(C-H) | aromatic hydrocarbones | (Hren et al., 2000) |
| 678 | *δ*(C-H) | aromatic hydrocarbones | (Hren et al., 2000) |
| 686 | *δ*(C-H) | aromatic hydrocarbones | (Hren et al., 2000) |
| 688 | *δ*(C-H) | aromatic hydrocarbones | (Hren et al., 2000) |
| 705 | *δ*(C-H) | aromatic hydrocarbones | (Hren et al., 2000) |
| 892 | *δ*(C＝C) | RR’C＝CH_2_ compounds | (Miskolczi et al., 2003) |
| 923 | *δ*(C-H) | carbohydrates | (Shen et al., 2016) |
| 925 | *δ*(C-H) | carbohydrates | (Shen et al., 2016) |
| 927 | *δ*(C-H) | carbohydrates | (Shen et al., 2016) |
| 929 | *δ*(C-H) | carbohydrates | (Shen et al., 2016) |
| 971 | aliphatic region | aliphatic alcohols or carbohydrates | (Jouraiphy et al., 2008) |
| 983 | *δ*(C＝C) | RCH＝CH_2_ compounds | (Miskolczi et al., 2003) |
| 1014 | aliphatic region | aliphatic alcohols or carbohydrates | (Jouraiphy et al., 2008) |
| 1060 | aliphatic region | aliphatic alcohols or carbohydrates | (Jouraiphy et al., 2008) |
| 1016 | C-O, Si-O | polysaccharides or silicates | (Tatzber et al., 2007) |
| 1064 | aliphatic region | aliphatic alcohols or carbohydrates | (Jouraiphy et al., 2008) |
| 1112 | aliphatic region | aliphatic alcohols or carbohydrates | (Jouraiphy et al., 2008) |
| 1159 | *ν*(C-O), *ν*(C-C) | carbohydrates | (Cael et al., 1975) |
| 1160 | *ν*(C-O), *ν*(C-C) | carbohydrates | (Cael et al., 1975) |
| 1166 | *ν*(C-O), *ν*(C-C) | carbohydrates | (Cael et al., 1975) |
| 1168 | *ν*(C-O), *ν*(C-C) | carbohydrates | (Cael et al., 1975) |
| 1180 | *ν*(C-O) | polyols | (Chandran et al., 2006) |
| 1184 | *ν*(C-O) | polyols | (Chandran et al., 2006) |
| 1186 | *ν*(C-O) | polyols | (Chandran et al., 2006) |
| 1187 | *ν*(C-O) | polyols | (Chandran et al., 2006) |
| 1189 | *ν*(C-O) | polyols | (Chandran et al., 2006) |
| 1193 | *ν*(C-O) | polyols | (Chandran et al., 2006) |
| 1191 | *ν*(C-O) | polyols | (Chandran et al., 2006) |
| 1203 | *ν*(C-O) | terpenes, carbohydrates | (Qi et al., 2016; Wang et al., 2011) |
| 1205 | *ν*(C-O) | terpenes, carbohydrates | (Qi et al., 2016; Wang et al., 2011) |
| 1207 | *ν*(C-O) | terpenes, carbohydrates | (Qi et al., 2016; Wang et al., 2011) |
| 1209 | *ν*(C-O) | terpenes, carbohydrates | (Qi et al., 2016; Wang et al., 2011) |
| 1367 | *δ*(C-H) | cellulose | (Garside and Wyeth, 2003) |
| 1369 | *δ*(C-H) | cellulose | (Gerçel et al., 2007; Garside and Wyeth, 2003) |
| 2967 | *ν_as_*(C-H) | RR’CH_2_ compounds | (Zhao et., 2015) |
| 2969 | *ν_as_*(C-H) | RCH_2_ | (Zhao et., 2015) |
| 3492 | *ν*(O-H) | benzoates, triterpenoids | (Gao et al., 2010; Xu et al., 2007) |
| 3494 | *ν*(O-H) | benzoates, triterpenoids, phenols | (Gao et al., 2010; Xu et al., 2007; Ivanova and Singh, 2003) |
| 3496 | *ν*(O-H) | benzoates, triterpenoids | (Gao et al., 2010; Xu et al., 2007) |
| 3498 | *ν*(O-H) | benzoates, triterpenoids | (Gao et al., 2010; Xu et al., 2007) |
| 3515 | *ν*(O-H) | benzoates, triterpenoids | (Gao et al., 2010; Xu et al., 2007) |
| 3517 | *ν*(O-H) | benzoates, triterpenoids | (Gao et al., 2010; Xu et al., 2007) |

*ν*: stretching vibration; *ν_as_*: asymmetric stretching vibration; *δ:* bending vibration

**References**

Cael, J. J., Koenig, J. L., and Blackwell, J. (1975). Infrared and raman spectroscopy of carbohydrates. Part VI: normal coordinate analysis of Vamylose. Biopolymers 14, 1885–1903. doi: 10.1002/bip.1975.360140909

Chandran, S. P., Chaudhary, M., Pasricha, R., Ahmad, A., and Sastry, M. (2006). Synthesis of gold nanotriangles and silver nanoparticles using Aloe vera plant extract. Biotechnol. Prog. 22, 577–583. doi: 10.1021/bp0501423

Gao, L., Li, J., and Qi, J. (2010). Gentisides A and B, two new neuritogenic compounds from the traditional Chinese medicines Gentiana rigescens Franch. Bioorg. Med. Chem. 18, 2131–2134. doi: 10.1016/j.bmc.2010.02.004

Garside, P., and Wyeth, P. (2003). Identification of cellulosic fibres by FTIR spectroacopy. Stud. Conserv. 48, 269–275. doi: 10.1179/sic.2003.48.4.269

Gerçel, Ö., Özcan, A., Özcan, A. S., and Gerçel, H. F. (2007). Preparation of activated carbon form a renewable bio-plant of Euphorbia rigida by H2SO4 activation and its adsorption behavior in aqueous solutions. Appl. Surf. Sci. 253, 4843–4852. doi: 10.1016/j.apsusc.2006.10.053

Hren, B., Katona, K., Mink, J., Kohán, J., and Isaák, G. (2000). Long-path FTIR spectroscopic studies of air poluutants in the Danube refinery plant. Analyst 125, 1655–1659. doi: 10.1039/b002398l

Ivanova, D. G., and Singh, B. R. (2003). Nondestructive FTIR monitoring of leaf senescence and elicitin-induced changes in plant leaves. Biopolymers 72, 79–85. doi: 10.1002/bip.10297

Jouraiphy, A., Amir, S., Winterton, P., El Gharous, M., Revel, J. C., and Hafidi, M. (2008). Structural study of the fulvic fraction during composting of activated sludge-plant matter: element analysis, FTIR and 13C NMR. Bioresour. Technol. 99, 1066–1072. doi: 10.1016/j.biortech.2007.02.031

Kizil, R., Irudayaraj, J., and Seetharaman, K. (2002). Characterization of irradiated starches by using FT-Raman and FTIR spectroscopy. J. Agric. Food. Chem. 50, 3912–3918. doi: 10.1021/jf011652p Miskolczi, N., Bartha, L., Deák, G., and Jóver, B. (2003). Chemical recycling of waste polyethylene and polypropylene. *J. Hazard. Mater.* 45, 125–130.

Miskolczi, N., Bartha, L., Deák, G., and Jóver, B. (2003). Chemical recycling of waste polyethylene and polypropylene. *Pet. Coal.* 45, 125–130.

Shen, Y. X., Zhao, Y. L., Zhang, J., Zuo, Z. T., Wang, Y. Z., and Zhang, Q. Z. (2016). Study on different parts of wild and cultivated *Gentiana rigescens* with fourier transform infrared spectroscopy. *Guang Pu Xue Yu Guang Pu Fen Xi* 36, 667–671.

Tatzber, M., Stemmer, M., Spiegel, H., Katzlberger, C., Haberhauer, G., Mentler, A., et al. (2007). FTIR-spectroscopic characterization of humic acids and humin fractions obtained by advanced NaOH, Na4P2O7 and Na2CO3 extraction procedures. J. Plant. Nutr. Soil. Sci. 170, 522–529. doi: 10.1002/jpln.200622082

Wang, Y., Li, W., Wu, D., Wang, J., Wu, C., Liao, J., et al. (2011). *In vitro* activity of 2-methoxy-1,4-naphthoquinone and stimasta-7,22-diene-3β-ol from *Impatiens balsamina* L. against multiple antibiotic-resistant *Helicobacter pylori. Evid. Based. Compl. Alt*. 2011. doi: 10.1093/ecam/nep147

Xu, M., Wang, D., Zhang, Y., and Yang, C. (2007). Dammarane triterpenoids from the roots of Gentiana rigescens. J. Nat. Prod. 70, 880–883. doi: 10.1021/np070012z

Zhao, Y., Zhang, J., Jin, H., and Zhan, J. (2015). Discrimination of Gentiana rigescens from different origins by fourier transform infrared spectroscopy combined with chemometric methods. J. AOAC. Int. 98, 22–26. doi: 10.5740/jaoacint.13-395

Zhbankov, R. G., Andrianov, V. M., and Marchewka, M. K. (1997). Fourier transform IR and raman spectroscopy and structure of carbohydrates. J. Mol. Struct. 436, 637–654. doi: 10.1016/S0022-2860(97)00141-5
